# Hepatic Cryotherapy and Subsequent Hepatic Arterial Chemotherapy for Colorectal Metastases to the Liver

**DOI:** 10.1155/1998/49496

**Published:** 1998-12

**Authors:** Richard S. Stubbs, Majeed H. Alwan, Michael W. C. Booth

**Affiliations:** The Wakefield Clinic for Gastrointestinal Diseases Wakefield Hospital Wellington New Zealand

## Abstract

This paper presents an experience of thirty consecutive patients with hepatic colorectal metastases who were treated with hepatic cryotherapy and subsequent hepatic arterial infusion (HAI) chemotherapy using 5FU.

Patients with colorectal metastases confined to the liver but not suitable for resection, and with liver involvement of less than 50% were offered the treatment. Prospective documentation of all patients was undertaken with data being recorded on a computerised database.

Patients had a median of 6 (2–15) lesions with sizes ranging from 1–12 cm. There was no 30 day mortality. Postoperative complications developed in 8 patients but were followed by full recovery in all instances. Side effects from chemotherapy occured in 23% of cycles. Twenty seven patients have died. Median survival from the time of cryotherapy was 18.2 months (7–34), or 23months (9–44) from diagnosis of liver lesions.

Hepatic cryotherapy with subsequent arterial chemotherapy is safe and well tolerated. The results suggest survival of patients with colorectal hepatic metastases can be improved by the use of this modality of treatment.


*HPB Surgery*, 1998, Vol. 11, pp. 97–104
Reprints available directly from the publisher
Photocopying permitted by license only

© 1998 OPA (Overseas Publishers Association) N.V.
Published by license under
the Harwood Academic Publishers imprint,
part of The Gordon and Breach Publishing Group.
Printed in Malaysia.

### Hepatic Cryotherapy and Subsequent Hepatic Arterial Chemotherapy for Colorectal Metastases to the Liver

RICHARD S. STUBBS[a,*], MAJEED H. ALWAN[b] and MICHAEL W. C. BOOTH[c]

[a] *Gastrointestinal/Hepatobiliary Surgeon,* [b] *Gastrointestinal Surgeon,*
[c] *Surgical Fellow, The Wakefield Clinic for Gastrointestinal Diseases, Wakefield Hospital, Wellington, New Zealand*

*(Received 28 July 1997; In final form 25 November 1997)*

**This paper presents an experience of thirty consecutive patients with hepatic colorectal metastases who were treated with hepatic cryotherapy and subsequent hepatic arterial infusion (HAI) chemotherapy using 5 FU.**

**Patients with colorectal metastases confined to the liver but not suitable for resection, and with liver involvement of less than 50% were offered the treatment. Prospective documentation of all patients was undertaken with data being recorded on a computerised database.**

**Patients had a median of 6 (2–15) lesions with sizes ranging from 1–12 cm. There was no 30 day mortality. Postoperative complications developed in 8 patients but were followed by full recovery in all instances. Side effects from chemotherapy occured in 23% of cycles. Twenty seven patients have died. Median survival from the time of cryotherapy was 18.2 months (7–34), or 23 months (9–44) from diagnosis of liver lesions.**

**Hepatic cryotherapy with subsequent arterial chemotherapy is safe and well tolerated. The results suggest survival of patients with colorectal hepatic metastases can be improved by the use of this modality of treatment.**

*Keywords:* Colorectal cancer, hepatic cryotherapy, liver metastases, hepatic artery chemotherapy

### INTRODUCTION

Colorectal cancer is a common disease in New Zealand with approximately 2000 new cases being registered annually. Liver metastases are found at diagnosis in some 25% of patients with colorectal cancer [1] and another 25–40% will eventually develop metastases [2]. Median survival for patients with untreated colorectal hepatic metastases is 6 to 8 months from diagnosis, and less than 2% survive for 5 years [3]. Surgical resection of liver metastases confers a median survival of over two years and a 25–52% five-year survival rate [4–6]. However, because of the number and/or location of metastases within the liver, fewer than 25% of patients are candidates for resection [7]. Cryotherapy with *in-situ* destruction of tumours is a possible treatment option. As a focal treatment, it spares more normal liver tissue than resection, thus allowing multiple lesions affecting both lobes of the liver to be treated.

*Corresponding author.

Good results have been achieved in patients with hepatocellular carcinoma [8] and encouraging results have been reported for patients with colorectal liver metastases [9 – 11].

Systemic chemotherapy regimens have been used for many years to treat metastatic colorectal cancer [12, 13]. The most active agent in this context is 5 fluorouracil (5 FU). The addition of folinic acid, which augments the cytotoxic activity of 5 FU and spares some toxicity, produces higher response rates than 5 FU alone. However, response rates remain less than 20% at the cost of significant systemic toxicity and there is little or no impact on survival [13, 14]. Hepatic arterial infusion (HAI) chemotherapy, in contrast, appears to be a more effective modality of treatment for non-resectable metastatic disease confined to the liver [15, 16]. In most studies, well over one third of the patients respond to this chemotherapy, and a median survival of 12 – 25 months has been reported [16 – 18]. A treatment protocol combining the potential benefits of hepatic cryotherapy and hepatic arterial 5 FU has been employed at The Wakefield Clinic since 1991 to treat patients with non-resectable colorectal hepatic metastases. This report presents the experience in the first 30 consecutive patients so treated.

### MATERIALS AND METHODS

Thirty patients with colorectal liver metastases underwent treatment between October 1991 and October 1994 at The Wakefield Clinic. They included 22 males and 8 females with a median age of 55 years (range 24 – 78). Preoperative investigations included chest X-ray, abdominal CT Scan, routine blood tests and CEA. Patients were selected on the following criteria: (1) they had metastatic colorectal cancer confined to the liver, (2) the disease was unsuitable for resection because of the number and/or distribution of lesions within the liver, (3) they were considered fit to undergo a major surgical procedure and (4) the disease was manageable with regard to the number of lesions (up to 10 – 15), their size (not more than one lesion > 5 cm), and less than 50% of the liver was involved. The treatment was carried out within a few weeks of evaluation at a time when most of the patients were asymptomatic. The liver metastases were synchronous in 24 patients and metachronous in 6. The original bowel tumour was Dukes C in 23 patients and Dukes B in 7. A median number of 6 lesions per patient were treated (range 2 – 15), and the size of lesions ranged from 1 – 12 cm.

The abdomen was explored through a bilateral subcostal incision under general anaesthetic and epidural. The liver and upper abdomen were thoroughly examined by palpation and intraoperative ultrasound (IOUS). Cholecystectomy was routinely carried out. Cryotherapy was performed using an LCS II system (Cryotech Ltd, UK) with either a 5 mm or 9 mm needle probe or flat probe through which liquid nitrogen was circulated at −196°C. Intraoperative ultrasound was used to detect occult liver tumours, guide the cryoprobe into lesions and monitor the freezing process. The aim was to freeze each lesion completely with a 5 – 10 mm margin of surrounding liver tissue. An arterial Infuse – a port (Infusaid USA) was placed subcutaneously over the inferior anterolateral aspect of the right rib cage. The catheter tip was positioned in the gastroduodenal artery at its origin from the common hepatic artery. Complete hepatic perfusion was confirmed by injecting 2 – 5 mL methylene blue through the port. In the event of incomplete liver perfusion, a second hepatic artery was sought. If feasible this was anastomosed to the hepatic artery already identified. Alternatively a second catheter from a separate port was inserted into the anomalous artery by a side branch created by division of the artery and end-to-side re-anastomosis. However, we recently started simply ligating the second artery and noticed a complete hepatic perfusion after reinjecting methylene blue. The access port was flushed with heparinised saline

(100 IU/mL). Avoidance of extrahepatic perfusion was accomplished by ligating the proximal branches of the gastroduodenal artery. All patients received a single prophylactic dose of 1.0 g IV Rocephin® (Ceftriaxone) and most received intra-operative IV mannitol 10 – 15 g to encourage good urine output. Patients were monitored for 24 hours post-operatively in the Intensive Care Unit.

HAI chemotherapy was usually started within 4 weeks of surgery. Patients were generally maintained as outpatients for this procedure and were encouraged to pursue normal activities. The regimen entailed 50 mg Folinic acid bolus *via* the port, followed by 5 FU 1.0 g each 24 hours for 4 days delivered by a disposable, portable pump (Baxter Healthcare Ltd, Singleday infusor PC1071). Cycles were planned every 4 weeks for a total of six cycles. If after 6 cycles CT Scanning revealed residual hepatic disease, HAI was continued. Treatment was discontinued if unacceptable side effects developed, hepatic artery thrombosis occured, or CT Scanning revealed unequivocal major disease progression.

Patients were re-evaluated at 3 monthly intervals with CT Scan of the liver, chest X-ray and routine blood tests including CEA. Disease status was determined radiologically as either tumour progression (an increase in size or number of metastases), stable disease or tumour regression. Disease assessments were based on a comparison between sequential 3 monthly CT Scans. On this basis patients may have been regarded as having disease progression at one point in time, yet the extent of hepatic involvement may still have been less than that prior to cryotherapy.

Fisher Exact test was used for statistical analysis.

### RESULTS

The surgery was well tolerated in most patients. It entailed a median operating theatre time of 5 hours. The number of lesions treated per patient varied from 2 to 15 (median 6). Nineteen (63.3%) patients were thought to have complete cryotherapy (cryo destruction of all macroscopic tumours), whereas in 11 (36.7%) patients, cryotherapy was considered incomplete. Cracking of the ice-ball upon thawing occured with the larger lesions resulting in bleeding but this was controlled with pressure and suture. Intraoperative blood loss was in the range 100 – 7240 mL (median 1400 mL) and 17 (56.7%) patients required blood transfusion. Postoperative complications developed in 8 patients. These included pleural effusion in 2, renal failure in 1, liver abscess in 1, wound infection in 1, myocardial infarction and atrial fibrillation in 1, congestive heart failure in 1, and skin burn in another patient. All patients recovered fully and there was no 30 day mortality. The median hospital stay was 9.5 days (range 6 – 23).

Two patients did not receive (HAI) chemotherapy for technical reasons related to port placement. Of the other 28 patients, 9 received 6 cycles, 7 received more than 6 cycles (7 – 27), and 12 received less than 6 cycles. A total of 209 cycles were given. Side effects developed in 48 (23%) cycles. These were considered mild in 8.6%, moderate in 9.1%, and severe in 5.3%. Mild side effects consisted of lethargy and nausea. Moderate side effects entailed nausea with marked epigastric pain. These were controlled either with medications or by reducing the dose of chemotherapy. In only 2 patients was chemotherapy discontinued due to unacceptable side effects. No instances of systemic toxicity with bone marrow depression, stomatitis, or diarrhoea were observed.

Preoperative CEA measurement was carried out in 26 patients and in 23 of them (88.5%) it was elevated. At 3 months postoperative follow-up 14/22 patients (64%) showed a reduction in CEA levels. In 7 of these it returned to normal levels. At 6 months 10/20 patients (50%) continued to have reduced levels. Twenty five of the 27 patients who have died has rising CEA prior to their death. In the other 2 cases data were insufficient to permit comment. One of the three living patients has a normal CEA level 45 months after cryotherapy. The other two cases have rising levels. One of them was found to have a solitary recurrent lesion in the right lobe of the liver with no evidence of extrahepatic spread of the disease. Right hepatectomy was subsequently performed 22 months after cryotherapy of 3 lesions. Now she is disease free 13 months after hepatic resection, and 35 months from cryotherapy.

At 6 months follow-up 19 patients showed static disease or regression on CT Scanning. In one of these all treated lesions had disappeared. At 9 months hepatic disease progression was evident in 19 patients, but 11 continued to have regression or stable disease. Of the 19 with disease progression 11 (57.9%) had received less than the planned 6 cycles of arterial chemotherapy, whereas 2 of the 11 (18.1%) patients with no evidence of progression had received less than 6 cycles of arterial chemotherapy. This difference is not statistically significant. At 12 months follow-up 8 patients had died, 18 had disease progression, and 4 continued to have static disease. However, as previously mentioned,

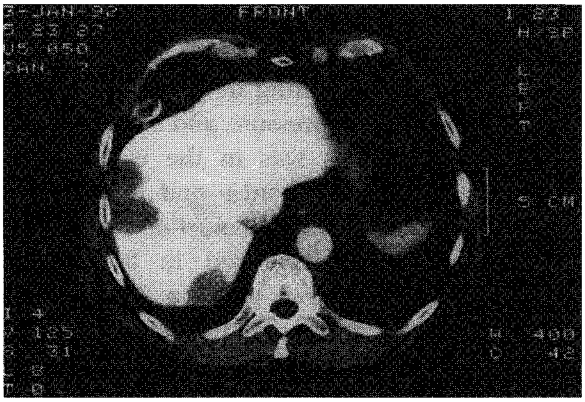

(a)

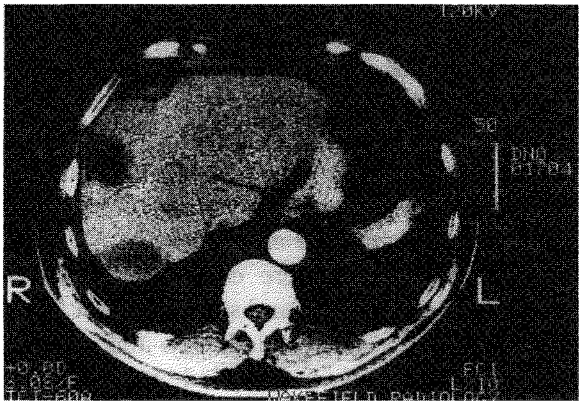

(b)

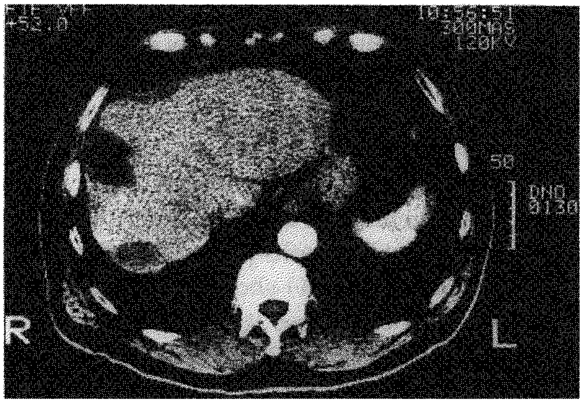

(c)

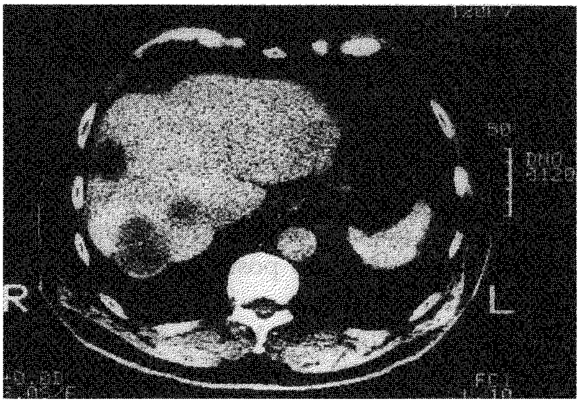

(d)

FIGURE 1 Series of CT Scans of a patient who had 9 metastatic liver lesions showing the changes in these following cryotherapy; (a) Peroperative CT Scan; (b) Postoperative scan at 6 months showing the lesions reducing in size; (c) Scan at 9 months showing the lesions were static; (d) Scan at 12 months showing the appearance of new lesions. When compared with previous scan, the disease was described as "progressed", however, hepatic involvement was still less than that of the original disease shown in (a).

patients who were regarded at one stage of follow-up as having disease progression, frequently still had less disease than that shown by CT Scan prior to cryotherapy. Figure 1a–d demonstrate the changes seen in one patient at different stages of follow-up.

At the time of death 8/27 patients (29.6%) had isolated liver disease as judged clinically and by chest X-ray and abdominal CT Scanning. In 4 of these hepatic arterial chemotherapy had been curtailed for various reasons. All other patients had abdominal disease (including local recurrence) and/or extra-abdominal disease. Extra-abdominal spread was to the lungs in all patients except in 3 who had bone metastases.

Eight patients died within 12 months of cryotherapy, 6 of whom (75%) received less than the planned 6 cycles of chemotherapy. Twenty two survived longer than 12 months after cryotherapy of whom seven (31.8%) received less than 6 cycles of chemotherapy. This difference is statistically significant ($P < 0.05$). There were no obvious differences in the nature and extent of disease prior to treatment in those who did or did not complete 6 cycles of chemotherapy.

Patients were followed till death or to November 1996. Median survival is 18.2 months (range 7–34) from cryotherapy or 23 months (range 9–44) from diagnosis of liver metastases. Twenty seven patients have died. Three patients are currently alive, 45, 42, and 35 months after treatment. One of them has a normal CEA and no evidence of tumour on abdominal CT Scan or chest X-ray 45 months after treatment of four metastatic sites within the liver.

### DISCUSSION

In patients with solitary liver lesions or lesions confined to one side of the liver, resection is the treatment of choice. A number of studies have demonstrated that this approach is not only safe but also potentially curative especially in patients with less than four metastases and a node-negative primary lesion [5, 19, 20]. In contradistinction to most types of liver metastases those from colorectal cancer can occur without tumour growth elsewhere [1, 21, 22].

Unfortunately, even in patients with metastases confined to the liver, the majority are not suitable for resection because of the number and/or distribution of these metastases within the liver. Systemic and regional chemotherapy are possible treatment options. In spite of moderately extensive world experience with hepatic arterial chemotherapy, the use of this modality has not gained general acceptance. There is reason however to believe it should be more widely considered for patients with metastatic colorectal cancer in the liver [17]. Regional perfusion delivers a high concentration of drug to the liver without incurring systemic dose-limiting toxicity. This is particularly so when drugs which are cleared on first pass through the liver such as 5 FU and fluorodeoxyuridine (FUDR) are employed. Prospective randomised trials comparing HAI chemotherapy with systemic chemotherapy have shown response rates of 48–62% with HAI compared to less than 20% for intravenous therapy [17, 18]. It is often stated that this improved response rate does not translate into survival advantage. However, there is evidence to suggest survival is improved [17, 18, 23]. All trials have confirmed the higher response rate of tumour to arterial chemotherapy and have shown an associated numerical survival advantage. In the largest study the survival advantage was statistically significant [24]. In two trials crossover between treatment groups was allowed and patients who did not respond to systemic chemotherapy were switched to HAI chemotherapy to which many responded [25, 26]. The role of adjuvant HAI therapy after liver resection has also been examined in a randomised trial [27]. Although the study involved only small numbers of patients, there was a trend toward increased survival in patients with resection plus HAI chemotherapy. In another single-arm study using adjuvant HAI chemotherapy after liver resection a lower incidence of recurrent disease was found when comparison was made with historical controls treated with surgery alone [28]. Thus the data suggest that local disease in the liver may be controllable by HAI.

A number of interstitial (focal) treatment modalities have been advocated for patients with hepatocellular carcinoma and hepatic metastases. These includes cryotherapy, alcohol injection, low power laser hyperthermia and interstitial radiotherapy [29]. In these modalities the therapeutic stimulus is delivered directly to a selected site of intended tissue damage, with preservation of the surrounding normal tissue. The effects on living tissue of sub-zero temperatures is reasonably well understood [30, 31] and if tempertures below −20°C are attained tissue destruction is almost always achieved. Cryotherapy of large solid tumours has been made possible following the development of appropriate delivery systems and probes utilising liquid nitrogen at −196°C. The LCS II (Cryotech, UK) is one such system currently available. The liver is an ideal organ for treatment by this modality because a margin of normal tissue can be destroyed with reasonable impunity. Furthermore intraoperative ultrasound can be employed to demonstrate the lesions within the liver and also the frozen "ice-ball" created by the freezing process. In this way a degree of precision can be applied to the treatment. The efficacy of the modality was first established following the report of the use of cryosurgery in hepatocellular carcinoma in which the results achieved in lesions less than 5 cm in diameter were comparable to surgical resection [8]. Subsequently a number of encouraging reports appeared in the literature concerning its use in the treatment of colorectal hepatic metastases [9–11].

In the present series, patients were not candidates for liver resection because they had a median of 6 hepatic lesions. Cryoablation was used to destroy these lesions and HAI chemotherapy was added in the hope of controlling remaining microscopic and on occasions macroscopic tumour.

The procedure was well tolerated with no 30 day mortality and few serious postoperative complications. Likewise side effects following arterial chemotherapy occured in only 23% of cycles. These were relatively minor and easily controlled with medications or by reducing the dose of chemotherapy. These side effects were due to local effects of chemotherapy reaching the upper gut from small unrecognised arteries supplying the stomach, duodenum or pancreas. No systemic toxicity was seen in any patient. Cholecystectomy was routinely performed to prevent chemical cholecystitis [18].

Initial control of the hepatic disease was indicated by the fact that 64% of patients showed a reduction in CEA levels at 3 months postoperative follow-up. In 7 of them it returned to normal. At 6 months follow-up 50% of them continued to have reduced levels. Failure to achieve reduction in CEA levels may be due to the incomplete cryotherapy in some patients or to occult hepatic or extrahepatic disease. Control of the disease was also noticed by follow up CT Scanning. At 6 months, only 11 patients had disease progression. At 9 months, 19 patients had disease progression and at 12 months follow-up, the disease was progressive in 26 patients. However, as previously mentioned, patients who were regarded at one stage of follow up as having disease progression, frequently still had less disease than that shown by CT Scan prior to cryotherapy (Fig. 1).

Eight patients died within 12 months of cryotherapy, 6 of whom (75%) received less than the planned 6 cycles of chemotherapy. Twenty two survived longer than 12 months after cryotherapy of whom 7 (31.8%) received less than 6 cycles of chemotherapy. This difference is statistically significant ($P < 0.05$) suggesting the addition of HAI chemotherapy confered additional benefit over cryotherapy alone. As mentioned previously there were no obvious differences in the nature and extent of disease prior to treatment in those who did or did not complete 6 cycles of chemotherapy.

From the data presented some control of liver metastases was achieved. The median survival of 18.2 months from cryotherapy or 23 months from diagnosis is better than reported median survival of 6–8 months for untreated patients with similar disease [3, 19]. These results are encouraging and indicate that this combination of treatment modalities merits further use and investigation in patients with colorectal hepatic metastases who are otherwise unsuitable for resection.

### References

[1] Bengmark, S. and Hafstorm, L. (1969). The natural history of primary and secondary malignant tumours of the liver: The prognosis for patients with hepatic metastases from colonic and rectal carcinoma by laparotomy. *Cancer*, **23**, 198–202.

[2] Bozzetti, F., Doci, R., Bignami, P., Morabito, A. and Gennari, L. (1987). Patterns of failure following surgical resection of colorectal cancer liver metastases. *Annals of Surgery*, **205**, 264–270.

[3] Bengtsson, G., Carlsson, G., Hafstorm, L. and Jonsson, P. E. (1981). Natural history of patients with untreated liver metastases from colorectal cancer. *American Journal of Surgery*, **141**, 586–589.

[4] Iwatsuki, S., Shaw, B. W. and Starzl, T. E. (1983). Experience with 150 liver resections. *Annals of Surgery*, **197**(3), 247–253.

[5] Doci, R., Gennari, L., Bignami, P., Montalto, A. and Bozzetti, F. (1991) One hundred patients with hepatic metastases from colorectal cancer treated by resection: Analysis of prognostic determinants. *British Journal of Surgery*, **78**, 797–801.

[6] Scheele, J., Stangl, R., Altendorf-Hofmann, A. and Gall, F. P. (1991). Indicators of prognosis after hepatic resection for colorectal secondaries. *Surgery*, **110**, 13–29.

[7] Foster, J. (1984). Treatment of metastatic disease of the liver: A skeptic's view. *Seminars in Liver Diseases*, **4**(2), 170–179.

[8] Zhou, X. D., Tang, Z. Y., Yu, Y. Q. and Ma, Z. C. (1988). Clinical evaluation of cryosurgery in the treatment of primary liver cancer: report of 60 cases. *Cancer*, **61**, 1889–1892.

[9] Onik, G., Rubinsky, B., Zemel, R., Weaver, L., Diamond, D., Cobb Charles and Porterfield, B. (1991). Ultrasound-guided hepatic cryosurgery in the treatment of metastatic colon carcinoma, Preliminary results. *Cancer*, **67**, 901–907.

[10] Ravikumar, T. S., Kane, R., Cady, B., Jenkins, R., Clouse, M. and Steele, G.JR. (1991). A 5 year study of cryosurgery in the treatment of liver tumours. *Archives of Surgery*, **126**, 1520–1524.

[11] Morris, D. L., Horton, M. D., Dilley, A. V., Warlters, A. and Clingan, P. R. (1993). Treatment of hepatic metastases by cryotherapy and regional cytotoxic perfusion. *Gut*, **34**, 1156–1157.

[12] Baker, L. H., Talley, R. W., Matter, R., Lehane, D. E., Ruffner, B. W., Jones, S. E., Morrison, F. S., Stephens, R. L., Gehan, E. A. and Vaitkevicius, V. K. (1976). Phase III comparison of the treatment of advanced gastrointestinal cancer with bolus weekly 5 FU *vs.* methyl-CCNU plus bolus weekly 5 FU. *Cancer*, **38**, 1–7.

[13] Petrelli, N., Douglass, H. O., Herrera, L., Russell, D., Stablein, D. M., Bruckner, H. W. *et al.* (24 authors) (1989). The modulation of fluorouracil with leucovorin in metastatic colorectal carcinoma: A prospective randomized phase III trial. *Journal of Clinical Oncology*, **7**, 1419–1426.

[14] Laufman, L. R., Bukowski, R. M., Collier, M. A., Sullivan, B. A., McKinnis, R. A., Clendennint, N. J., Guaspari, A. and Brenckman, W. D. (1993). A randomized, double-blind trial of fluorouracil plus placebo *versus* fluorouracil plus oral leucovorin in patients with metastatic colorectal cancer. *Journal of Clinical Oncology*, **11**, 1888–1893.

[15] Niederhuber, J. E., Esminger, W., Gyves, J., Thrall, J., Walker, S. and Cozzi, E. (1984). Regional chemotherapy of colorectal cancer metastatic to the liver. *Cancer*, **53**, 1336–1343.

[16] Goldberg, J. A., Kerr, D. J., Wilmott, N., McKillop, J. H. and McArdle, C. S. (1990). Regional chemotherapy for colorectal liver metastases: a phase II evaluation of targeted hepatic arterial 5 fluorouracil for colorectal metastases. *British Journal of Surgery*, **77**, 1238–1240.

[17] Kemeny, N. (1992). Is hepatic infusion of chemotherapy effective treatment for liver metastases? Yes! In Important Advances in Oncology, Edited by De Vita, V. T., Hellman, S. and Rosenberg, S. A., pp. 207–227, Philadelphia: J. B. Lippincott.

[18] McCall, J. L., Jorgensen, J. O. and Morris, D. L. (1995). Hepatic artery chemotherapy for colorectal liver metastases. *The Australian and New Zealand Journal of Surgery*, **65**, 383–389.

[19] Adson, M. A., van Heerden, J. A., Adson, M. H., Wagner, J. S. and Ilstrup, D. M. (1984). Resection of hepatic metastases from colorectal cancer. *Archives of Surgery*, **119**, 647–651.

[20] Hughes, K. S., Simon, R., Songhorabodi, S., Adson, M. A., Ilstup, D. M., Fortner, J. G. *et al.* (49 authors) (1986). Resection of the liver for colorectal carcinoma metastases: A multi-institutional study of patterns of recurrence, *Surgery*, **100**, 278–284.

[21] Steele, G.JR. and Ravikumar, T. S. (1989). Resection of hepatic metastases from colorectal cancer. Biologic perspectives. *Annals of Surgery*, **210**, 127–138.

[22] Ekberg, H., Tranberg, K. G., Andersson, R., Lundstedt, C., Hagerstrand, I., Ranstam, J. and Bengmark, S. (1987). Pattern of recurrence in liver resection for colorectal secondaries. *World Journal of Surgery*, **11**, 541–547.

[23] Allen-Marsh, T. G., Earlam, S., Fordy, C., Abrams, K. and Houghton, J. (1994). Quality of life and survival with continuous hepatic-artery floxuridine infusion for colorectal liver metastases. *The Lancet*, **344**, 1255–1260.

[24] Rougier, P., Laplanche, A., Huguier, M., Hay, J. M., Ollivier, J. M., Escat, J. *et al.* (18 authors) (1992). Hepatic arterial infusion of floxuridine in patients with liver metastases from colorectal carcinoma: Long-term results of a prospective, randomized trial. *Journal of Clinical Oncology*, **10**, 1112–1118.

[25] Kemeny, N., Daly, J., Reichman, B., Geller, N., Botet, J. and Oderman, P. (1987). Intrahepatic or systemic infusion of fluorodeoxyuridine in patients with liver metastases from colorectal carcinoma: A randomized trial. *Annals of Internal Medicine*, **107**, 459–465.

[26] Hohn, D. C., Stagg, R. J., Friedman, M. A., Hannigan, J. F. Jr., Rayner, A., Ignoffo, R. J., Acord, P. and Lewis, B. J. (1989). A randomized trial of continuous intravenous *vs*. hepatic intra-arterial floxuridine in patients with colorectal cancer metastatic to the liver: The Northern California Oncology Group trial. *Journal of Clinical Oncology*, **7**, 1646–1654.

[27] Kemeny, M. M., Goldberg, D., Beatty, J. D., Blaney, D., Browning, S., Doroshow, J., Genteaume, L., Hill, R., Kokal, W. A., Rihimaki, D. U. and Terz, J. J. (1986). Results of a prospective randomized trial of continuous regional chemotherapy and hepatic resection as treatment of hepatic metastases from colorectal primaries. *Cancer*, **57**, 492–498.

[28] Curley, S. A., Roh, M. S., Chase, J. L. and Hohn, D. C. (1993). Adjuvant hepatic arterial infusion chemotherapy after curative resection of colorectal liver metastases. *American Journal of Surgery*, **166**, 743–748.

[29] Masters, A., Steger, A. C. and Bown, S. G. (1991). Role of interstitial therapy in the treatment of liver cancer. *British Journal of Surgery*, **78**, 518–523.

[30] Cooper, I. S. (1963). Cryogenic surgery: a new method of destruction or extirpation of benign or malignant tissues. *New England Journal of Medicine*, **268**, 743–749.

[31] Zacarian, S. A. and Adham, M. I. (1966). Cryotherapy of cutaneous malignancy. *Cryobiology*, **2**, 212–216.

